# Hyperammonemia in a carbamoyl-phosphate synthetase 1 deficiency recipient after living-donor liver transplantation from a carrier donor: a case report

**DOI:** 10.3389/fmed.2023.1327854

**Published:** 2024-01-03

**Authors:** Toshihiko Kakiuchi, Tetsuya Nosho, Masafumi Oka, Katsuya Tashiro

**Affiliations:** ^1^Department of Pediatrics, Faculty of Medicine, Saga University, Saga, Japan; ^2^Department of Pediatrics, Karatsu Red Cross Hospital, Karatsu, Japan

**Keywords:** carbamoyl-phosphate synthetase 1 deficiency disease, hyperammonemia, liver transplantation, ornithine transcarbamylase deficiency disease, heterozygous

## Abstract

Carbamoyl-phosphate synthetase 1 (CPS1) deficiency is an autosomal recessive congenital urea cycle disorder (UCD) characterized by hyperammonemia. The recipients of liver transplantation (LT) for UCD are often children, and the potential donors are often the parents. Hereditary congenital diseases involving UCD entail the possibility of both parents being genetically heterozygous. Herein, we describe the case of a 12-year-old girl with CPS1 deficiency receiving a liver transplant (soon after birth) from her father, who had a heterozygous *CPS1* mutation. She was referred to our hospital with respiratory distress after contracting two infections (respiratory syncytial virus and human metapneumovirus) within a short period, both of which presented with hyperammonemia. Medication for hyperammonemia quickly lowered the ammonia levels. The hyperammonemia was thought to be caused by the heterozygous mutation in the donor liver; moreover, it is likely that the low enzyme activity in the patient’s liver was increased due to the infections. This is the first study to report hyperammonemia in a CPS1 deficiency patient due to an infection after LT. Thus, patients with CPS1 deficiency should be aware of the development of hyperammonemia after LT.

## 1 Introduction

Carbamoyl-phosphate synthetase 1 (CPS1) deficiency is an autosomal recessive congenital urea cycle disorder (UCD) characterized by hyperammonemia, with a morbidity rate of 1/50,000 to 1/800,000 (1–3). The initial treatment for this CPS1 deficiency-based hyperammonemia involves protein restriction and supplementation with arginine, sodium benzoate, sodium phenylacetate/phenylbutyrate, L-citrulline, and carnitine (4). However, these treatments may not successfully avoid ammonia accumulation and recurrent hyperammonemia, resulting in neurologic sequelae that could lead to death (5).

Liver transplantation (LT) is the final treatment for CPS1 deficiency patients who have difficulty withdrawing the continuous plasmapheresis for hyperammonemia (6, 7). Currently, LT is the only curative treatment option until novel therapies become available (8). The recipients of LDLT for UCD are often children, and the potential donors are often the parents.

Herein, we report a case of hyperammonemia in a patient with CPS1 deficiency who received a liver donation from a heterozygous father.

## 2 Case report

A 12-year-old bedridden girl was referred to our hospital with respiratory distress. She was born healthy at 39 weeks gestation, but developed a fever from the second day after birth and was admitted to a secondary hospital, where she developed respiratory failure, jaundice, and convulsions, with ammonia levels > 400 μg/dL. The next day, she was referred to a tertiary care hospital, where continuous hemodiafiltration was immediately introduced. The ammonia level rose to a maximum of 2,700 μg/dL and declined rapidly, reaching near-normal levels after 2 days. Laboratory findings showed elevated plasma glutamine and alanine levels and decreased arginine and citrulline levels, with no increase in urinary orotic acid excretion; consequently, a biochemical diagnosis of CPS1 deficiency was reached. A genetic test showed a compound heterozygous pathogenic variant in the *CPS1* gene (OMIM: 237300; c.1529del [p.Gly510AlafsTer5] in exon 34 and c.2339G>A [p.Arg780His] in exon 34; Table 1). Intensive care was provided, but the patient remained bedridden due to hyperammonitic encephalopathy. At 6 months of age, the patient underwent living-donor liver transplantation (LDLT) from her father. Her liver function was stabilized, and she was followed up at a secondary hospital with nutritional therapy for a long time.

**Table 1:** The genetic test results of the patient (recipient) and her father (liver donor).

		Gene name	Feature ID	Genotype	Annotation	HGVS. C	HGVS. P	Position
Patient	Recipient	CPS1	NM_001875. 5	Heterozygous	Frameshift variant	c. 1529del	p. Gly510AlafsTer5	chr2:210599539
		CPS1	NM_001875. 5	Heterozygous	Missense variant	c. 2339G>A	p. Arg780His	chr2:210608507
Father	Donor	CPS1	NM_001875. 5	Heterozygous	Missense variant	c. 2339G>A	p. Arg780His	chr2:210608507

CPS1, Carbamoyl-phosphate synthetase 1; HGVS, Human Genome Variation Society (c, coding; p, protein).

**Figure 1:**
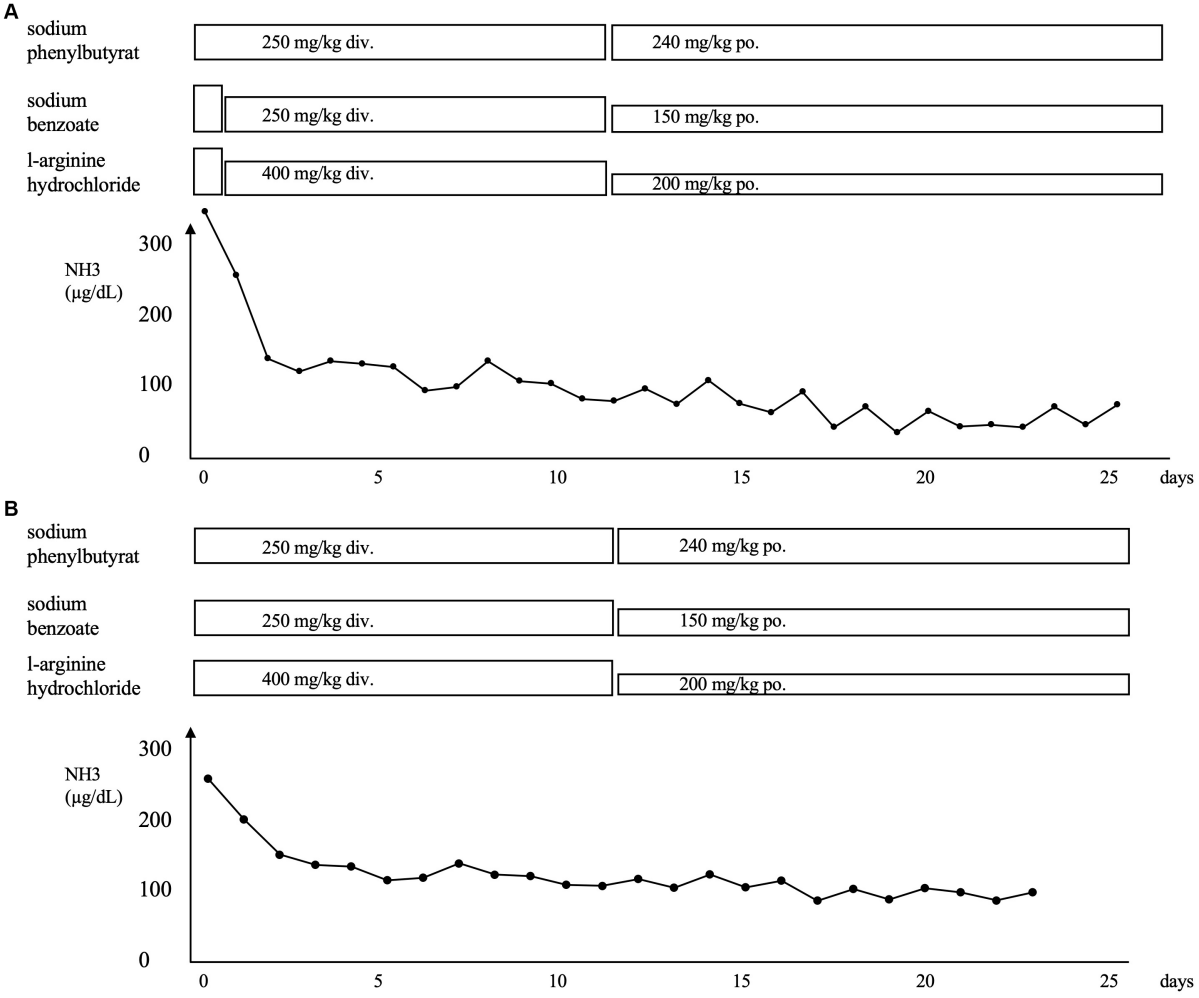
**(A)** Clinical course of the patient after the first admission to our hospital for respiratory syncytial virus infection. **(B)** Clinical course of the patient after admission to our hospital for human metapneumovirus infection. The administration of sodium phenylbutyrate, l-arginine hydrochloride, and sodium benzoate was initiated, and a rapid improvement in hyperammonemia was observed in both instances. NH_3_, ammonia; div., drip infusion into vein; po., *per os*.

About 5 days ago, the patient was referred to our hospital with wheezing and retractions after contracting an infection with respiratory syncytial virus (RSV). Despite treatments at the referral hospital, the hypercapnia progressed. The laboratory data after admission to our hospital were as follows: ammonia, 324 μg/dL; pH, 7.170; pCO_2_, and 84.0 mmHg (Figure 1A). She was placed on mechanical ventilation, which rapidly resolved the hypercapnia. Administration of sodium phenylbutyrate (250 mg/kg), l-arginine hydrochloride (400 mg/kg), and sodium benzoate (250 mg/kg) was initiated, and the ammonia level was reduced to 130 μg/dL the next day. The respiratory condition remained stable thereafter. The ammonia level remained at the upper limit of the normal range, and the patient was transferred to her previous hospital on the 26th day of admission to our hospital.

Four months later, she developed another infection with human metapneumovirus with a worsening of the respiratory condition like the last time. Figure 1B shows the clinical course of the patient. The laboratory data were as follows: ammonia, 265 μg/dL; pH, 7.301; and pCO_2_, 103.0 mmHg. She was placed on mechanical ventilation, and the hyperammonemia was treated with the same medication as before via intravenous administration. The ammonia level dropped to 128 μg/dL the next day and gradually decreased until it returned to normal 2 weeks later. The patient was transferred to the previous hospital on the 23rd day after admission. Oral medication for high ammonia was continued. The patient was promptly discharged from the other hospital and has had no infections or hyperammonemia since then.

Genetic testing of the father showed a heterozygous pathogenic variant in the *CPS1* gene (OMIM: 237300; c.2339G>A [p.Arg780His]) in exon 34 (Table 1), which matched that in the patient.

## 3 Discussion

The clinical course of the female patient in this study highlights that careful attention must be paid regarding the development of hyperammonemia after LTDT, particularly in cases where the donor is a relative with a heterozygous mutation for CPS1 deficiency.

LDLT for metabolic disorders is the key option in Japan. Most metabolic disorders have an autosomal recessive inheritance pattern. Parents who are obligate carriers of the recipient’s disorders become potential heterozygous donors (9). LDLT from heterozygous donors is a feasible option with a better quality of life in patients with CPS1 deficiency (10). On the other hand, in those with OTC deficiency, a transplant from an OTC deficiency heterozygous carrier should be avoided if another donor candidate is available due to the development of the potentially fatal hyperammonemia following LDLT (11). Wakiya et al. (12) proposed donor determination based on the ornithine transcarbamylase activity of the donor liver before LT.

To our knowledge, the present case is the first to report the development of hyperammonemia in a CPS1 deficiency patient due to an infection after LT. However, there are two unresolved issues in this case study. The first is that the causes of the two episodes of hyperammonemia were not wholly determined. The CPS1 enzyme activity in the donor liver was likely lower than normal, and the ability to process ammonia was decreased due to the infection. The father had never exhibited any symptoms suggesting hyperammonemia during infection. However, the enzyme activity levels in the donor livers were not measured in his life. Second, the types and severity of infections that cause hyperammonemia remain unclear. The patient experienced fever due to respiratory infections and aspiration pneumonia in the past but had never presented with hyperammonemia. This point is crucial as we continue to follow up on the patient; otherwise, the hyperammonemia may be overlooked, and medical overreach may result in frequent ammonia measurements.

In conclusion, as with OTC deficiency after LT, patients with CPS1 deficiency should be aware of the development of hyperammonemia at any time.

## Data availability statement

The original contributions presented in the study are included in the article/supplementary material, further inquiries can be directed to the corresponding author.

## Ethics statement

Written informed consent was obtained from the minor(s)' legal guardian/next of kin for the publication of any potentially identifiable images or data included in this article.

## Author contributions

TK: Conceptualization, Data curation, Investigation, Methodology, Project administration, Supervision, Writing – original draft, Writing – review & editing. TN: Data curation, Investigation, Writing – review & editing. MO: Data curation, Investigation, Writing – review & editing. KT: Conceptualization, Data curation, Investigation, Project administration, Writing – review & editing.
